# The Two Cis-Acting Sites, *parS1* and *oriC1*, Contribute to the Longitudinal Organisation of *Vibrio cholerae* Chromosome I

**DOI:** 10.1371/journal.pgen.1004448

**Published:** 2014-07-10

**Authors:** Ariane David, Gaëlle Demarre, Leila Muresan, Evelyne Paly, François-Xavier Barre, Christophe Possoz

**Affiliations:** 1 CNRS, Centre de Génétique Moléculaire, Gif-sur-Yvette, France; 2 Université Paris-Sud, Orsay, France; Institute of Molecular and Cell Biology (IMCB), A*STAR, Singapore

## Abstract

The segregation of bacterial chromosomes follows a precise choreography of spatial organisation. It is initiated by the bipolar migration of the sister copies of the replication origin (*ori*). Most bacterial chromosomes contain a partition system (Par) with *parS* sites in close proximity to *ori* that contribute to the active mobilisation of the *ori* region towards the old pole. This is thought to result in a longitudinal chromosomal arrangement within the cell. In this study, we followed the duplication frequency and the cellular position of 19 *Vibrio cholerae* genome loci as a function of cell length. The genome of *V. cholerae* is divided between two chromosomes, chromosome I and II, which both contain a Par system. The *ori* region of chromosome I (*ori_I_*) is tethered to the old pole, whereas the *ori* region of chromosome II is found at midcell. Nevertheless, we found that both chromosomes adopted a longitudinal organisation. Chromosome I extended over the entire cell while chromosome II extended over the younger cell half. We further demonstrate that displacing *parS* sites away from the *ori_I_* region rotates the bulk of chromosome I. The only exception was the region where replication terminates, which still localised to the septum. However, the longitudinal arrangement of chromosome I persisted in Par mutants and, as was reported earlier, the *ori* region still localised towards the old pole. Finally, we show that the Par-independent longitudinal organisation and *ori_I_* polarity were perturbed by the introduction of a second origin. Taken together, these results suggest that the Par system is the major contributor to the longitudinal organisation of chromosome I but that the replication program also influences the arrangement of bacterial chromosomes.

**Author summary**

Proper chromosome organisation within the cell is crucial for cellular proliferation. However, the mechanisms driving bacterial chromosome segregation are still strongly debated, partly due to their redundancy. Two patterns of chromosomal organisation can be distinguished in bacteria: a transversal chromosomal arrangement, such as in *E. coli*, where the origin of replication (*ori*) is positioned at midcell and flanked by the two halves of the chromosome (replichores), and a longitudinal arrangement, such as in *C. crescentus*, where *ori* is recruited to the pole and the replichores extend side by side along the long axis of the cell. Here, we present the first detailed characterization of the arrangement of the genetic material in a multipartite genome bacterium. To this end, we visualised the position of 19 loci scattered along the two *V. cholerae* chromosomes. We demonstrate that the two chromosomes, which both harbour a Par system, are longitudinally organised. However, the smaller one only extended over the younger cell half. In addition, we found that disruption of the Par system of chromosome I released its origin from the pole but preserved its longitudinal arrangement. Finally, we show that the addition of an ectopic *ori* perturbed this arrangement, suggesting that the replication program contributes to chromosomal organisation.

## Introduction

Bacterial chromosome replication is initiated from a unique origin (*oriC*) and progresses bidirectionally. The replication of circular chromosomes terminates opposite the *oriC* in a region termed the terminus (Ter). Within the Ter region is a site-specific recombination site, termed *dif*, dedicated to the resolution of chromosome dimers [1]. These factors define two replication arms, Left and Right, mirrored by the <*oriC*-*dif*> axis. Detailed investigations of the choreography of chromosomal movements during the cell cycle of several monochromosomic bacteria suggest that segregation is concurrent with replication and starts with the precise positioning of newly replicated sister copies of the *oriC* region into opposite cell halves [2], [3], [4], [5]. Segregation of other sister chromosomal loci to their positions in daughter cells follows shortly after their replication with sister copies of Ter being segregated last [2], [3], [4], [5]. Less is known about the choreography of chromosome segregation in bacteria with multipartite genomes. However, the analysis of a single locus in the *oriC* region and a single locus in the putative Ter region of the two *Vibrio cholerae* chromosomes suggests a model of replication and segregation that is consistent with monochromosomic bacteria [6], [7], [8]. Taken together, these observations suggest that the active positioning of the *oriC* region sets the pace for chromosome segregation, raising questions regarding the underlying mechanism.

Similar to most other bacteria, a specific partition system is encoded on each of the *Vibrio cholerae* chromosomes [9]. Bacterial chromosome partition machineries are related to the Type I partitioning systems of plasmids. They consist of two genes, *parA*, which codes for an ATPase, and *parB*, which codes for a sequence-specific DNA binding protein that is able to spread around its binding site, *parS* [10]. Several *parS* sites are usually found proximal to and in some cases encompassing the *oriC* region of bacterial chromosomes [9]. The role of Par systems in DNA segregation is well established for low-copy number plasmids [11], [12], [13]. However, their role in bacterial chromosome segregation remains controversial, notably because the disruption of Par systems in different bacterial species produces different phenotypes. The Par systems of *Caulobacter crescentus*, *Myxococcus xanthus* and *V. cholerae* chromosome II are essential for chromosome segregation [4], [14], [15]. The impairment of the *Pseudomonas aeruginosa* Par system caused the formation of ∼20% anucleate cells [16]. However, the disruption of the Par machinery affects few cells (<2%) in *Bacillus subtilis* [17] and yields no segregation defect for *V. cholerae* chromosome I [18]. Moreover, several bacterial species, notably *Escherichia coli* and *Streptococcus pneumonia*, lack a functional Par system. Partition systems have also been implicated in other cellular processes including replication initiation [19], [20], cell cycle coordination [4], [14] and chromosome compaction [21], [22], [23]. Finally, ParB-binding to *oriC*-proximal *parS* sites recruits SMC proteins to the origin region in *B. subtilis* and *S. pneumoniae*, contributing to chromosome segregation even in the absence of ParA [21], [22], [23]. Nevertheless, Par systems are directly involved in the polar positioning and the active bipolar migration of the *oriC* region of the *C. crescentus* chromosome and *V. cholerae* chromosome I [8], [24], [25], [26], [27]. The polar anchoring mechanisms have been described for these two systems [27], [28].

The characterization of chromosomal organisation in different bacterial species suggests a common mechanism of longitudinal organisation in which the *oriC* region is positioned towards the old pole, Ter is positioned towards the new pole, and the two chromosome replichores extend over the long axis of the cell [3], [4], [29], [30]. The polar localisation of the *oriC* regions of *V. cholerae* chromosome I and of the multiple chromosomes of *S. meliloti* and *A. tumefaciens* suggests a similar longitudinal organisation [31], [32]. In contrast, the *E. coli* chromosome, which seems devoid of a Par system, adopts a transversal organisation where the *oriC* region is positioned at midcell and the left and right chromosomal arms extended toward the opposite cell halves [33], [34]. Taken together, these observations suggest that it is the active positioning of the *oriC* region towards the old cell pole by Par systems that is responsible for the longitudinal chromosomal organisation observed in most bacterial species.

However, two characteristics of *V. cholerae* make it an ideal bacterial model to test this hypothesis. Firstly, the Par system of *V. cholerae* chromosome II positions the origin region (*oriC2*) at midcell rather than at the cell pole, which would be expected to drive a transversal arrangement if the hypothesis was correct. Secondly, disruption of the Par system of *V. cholerae* chromosome I does not affect any step of the cell cycle, allowing for the direct investigation of its contribution to chromosomal organisation. In this current study, the analysis of the intracellular location of 12 chromosome I loci and 7 chromosome II loci in exponentially growing cells indicated that both *V. cholerae* chromosomes adopted a longitudinal organisation. Chromosome I extends from the old pole to the new pole and chromosome II extends from midcell to the new pole, i.e. in the younger cell half. By displacing *parS1* sites away from *oriC* the Par system was shown to contribute to the longitudinal organisation of chromosome I. However, the *parS1-*deleted chromosome I remained longitudinal, with the *oriC* locus remaining located close to the old pole. The insertion of an ectopic origin of replication was sufficient to disrupt the longitudinal organisation of *parS1* deleted chromosome I. Interestingly, the ectopic origin of replication was often positioned closer to the old pole than the original *oriCI*. Taken together, these observations suggest that the replication program contributes to the longitudinal organisation of *V. cholerae* chromosome I.

## Results

### Fluorescent labelling systems and analysis of chromosome choreographies

Chromosome choreography involves successive steps of chromosome organisation as a function of cell cycle progression. To avoid any complications linked to multiple concurrent rounds of replication, cells were cultivated at 37°C in slow growing conditions (M9 Fructose supplemented with thiamine) characterised by a 55 min generation time divided into three successive periods: a period of 11 min before replication initiation (B period), a 32 min-long replication period (C period) and a 12 min period after replication and before division (D period) [35]. Snapshot images of cells in steady state exponential growth were acquired. By correlating the length of cells with their progression through the cell cycle we studied chromosomal organisation as a function of cell elongation. Cells were classified according to their length in 0.1 µm intervals. A sliding window of 0.3 µm was used to determine the median position and frequency of duplication of any specific chromosomal locus. In our growth conditions the majority of cells had a length between 1.9 µm and 4.3 µm. We will refer to the cells of the first point plotted, corresponding to the 2 µm interval, as newborn cells and the cells of the last interval plotted, corresponding to the 4.2 µm interval, as dividing cells.

The spatial organisation of chromosome I and of chromosome II was deduced from the positioning of 12 and 7 loci respectively. They were broadly distributed over the entire genome, particularly the regions of special interest, such as the origin of replication and the chromosomal dimer resolution site of each of the two chromosomes (*ori_I_, ori_II_, ter_I_ and ter_II_*). Five loci were tagged on the right (*R1_I_* to *R5_I_*) and left (*L1_I_* to *L5_I_*) replichores of chromosome I. On chromosome II, three loci were tagged on the right replichore (*R1_II_* to *R3_II_*) and two on the left (*L1_II_* and *L2_II_*). The loci were visualised in pairwise combinations using two compatible fluorescent labelling systems, a *lacO* array was inserted at one of the loci and a *parS*_pMT1_ site at the other locus. LacI-mCherry and yGFP-Δ30ParB_pMT1_ [34] protein fusions were produced from an operon in place of the *V. cholerae lacZ* gene. In some cases, a third locus was tagged with a *tetO* array and visualized by the production of a TetR-Cerulean fusion from a plasmid. We observed the same pattern of localisation of the *L3_I_* or *terI* loci during the cell cycle whether they were tagged with a *lacO* or *tetO* array or with a *parS*_pMT1_ site, suggesting that these three systems did not affect the positioning of the chromosomal loci under our experimental conditions.

Dual labelling has three advantages. Firstly, the precise orientation of the long axis of the cell from the new pole to the old pole could be determined. The new pole is defined as the pole resulting from the previous cell division event. Assuming no gross rearrangements of chromosomal DNA after cell division, a locus closer to the septum than to the poles in dividing cells was defined as being closer to the new pole than to the old pole in newborn cells. Time-lapse microscopy observations validated this method (Figure S1). Secondly, dual labelling allowed for the collection of data in two different strains even if they presented slightly different cell size distributions (Figure S2). The cell size distribution of the two strains was re-aligned using the frequency of duplication of a common locus as a reference. In the rest of the manuscript we will refer primarily to compiled figures. However, each of our conclusions could be drawn based on individual strain data. The data of each individual strain are presented in Figures S3 to S39. Thirdly, dual labelling allowed for the comparison of the timing of duplication of the two tagged loci of each strain as a function of cell elongation and the direct measure of the distance separating these loci as a function of cell elongation. These comparisons could be made independently of any orientation procedure or cell size re-alignment.

### Sequential order of duplication of chromosomal DNA in *V. cholerae*

**Figure 1:**
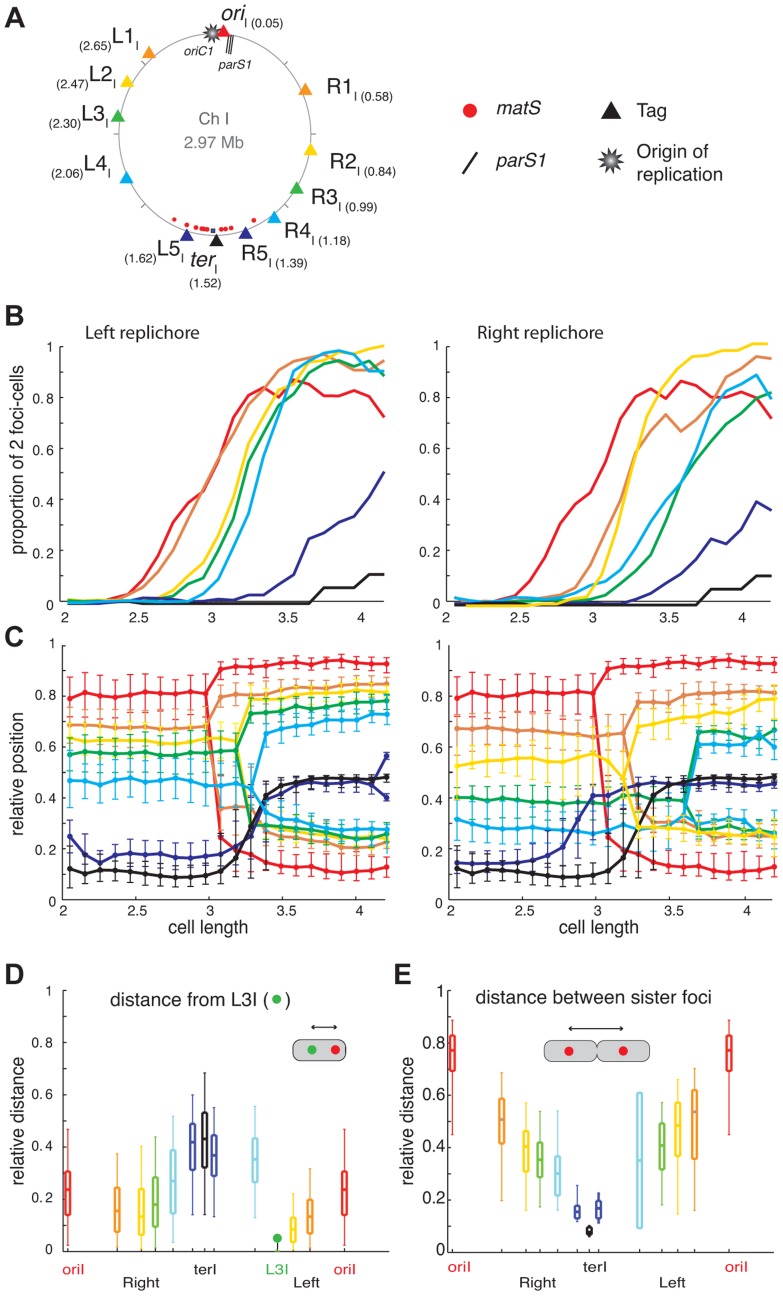
Longitudinal organisation of *V. cholerae* chromosome I: Sequential duplication and segregation. (A) Circular *V. cholerae* chromosome I and II maps indicating the position of the different tags with respect to *oriC1*, *parS1* and *matS* sites and their corresponding colour code. (B) Proportion of 2 foci cells according to the realigned cell length (cell size intervals of 0.1 µm) for the different loci of the chromosome I. For each locus, a minimum number of 800 cells were analysed. Left panel: left replichore; Right panel: right replichore. The first cell size interval where ≥50% of cells contained two *L3_I_* foci served to align the cell length distributions of ADV20, ADV21, ADV22, ADV23, ADV25, ADV33, ADV42, ADV50 and ADV51 strains, using ADV24 *L3_I_* as reference. The strain EPV213 was aligned against ADV42 using the timing of recruitment of *ter*_I_ to midcell. (C) Reconstitution of the segregation choreographies of the 12 chromosome I loci. Left panel: left replichore; Right panel: right replichore. The median, the 25^th^ and the 75^th^ percentiles of the relative cell position of each locus are plotted for each cell size interval. The cells falling into the first interval were named newborn cells and the ones falling into the last interval were named dividing cells. 0: new pole; 1: old pole. (D) The relative distance between different chromosome I loci to the L3I locus was measured as a function of the relative cell length in the cells containing only one focus of each locus. The median (horizontal bar), the 25th and the 75th percentiles (open box) and the 5th and the 95th percentiles (error bars) of the distance of a given locus to L3I were indicated at this locus position along a chromosome I linear genetic map. (E) Relative distance between any of the chromosome sister loci, measured in cells with a length >3.4 µm.

**Figure 2:**
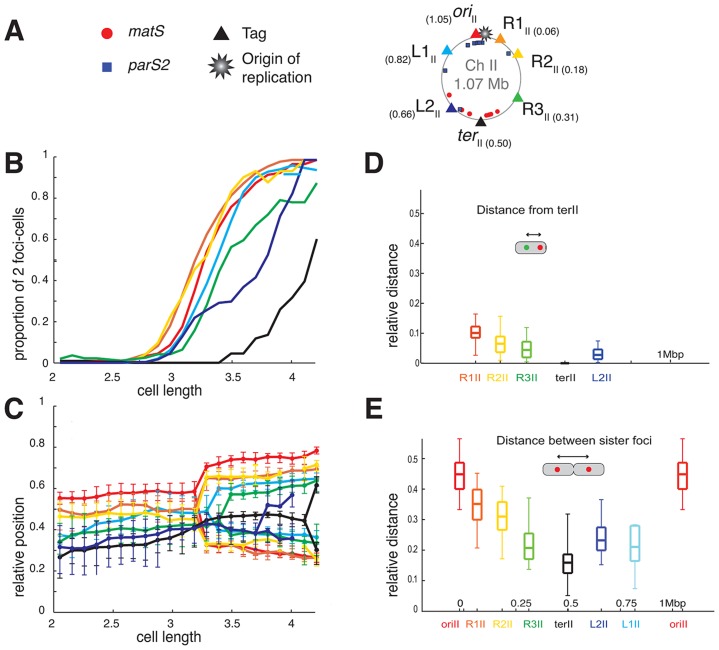
Longitudinal organisation of *V. cholerae* chromosome II: Sequential duplication and segregation. (A) Circular *V. cholerae* chromosome II map indicating the position of the different tags with respect to *oriC2*, *parS2* and *matS* sites and their corresponding colour code. (B) Proportion of 2 foci cells according to the realigned cell length (cell size intervals of 0.1 µm) for the different loci of chromosome II. For each locus, a minimum number of 800 cells were analysed. The first cell size interval where ≥50% of cells contained duplicated *L3_I_* was used to realigned ADV26 to the ADV24 reference strain. CP708, ADV131, ADV30 and ADV131 cell size distributions were aligned with the GDV552 cell sizes based on the cell size interval where ≥25% of cells contained two *ter_II_* loci. GDV552 was aligned to ADV42 using the cell size interval where *ter_I_* is recruited to midcell. ADV123 was aligned with ADV24 using the first cell size interval where ≥50% of cells contained two *ori_I_*. (C) Reconstitution of the segregation choreographies of the 7 chromosome II loci, as in Figure 1C. (D) Relative distance between any of the chromosome II loci to the *ter_II_* locus, measured in the cells containing only one focus of each locus. (E) Relative distance between any of the chromosome sister loci, as in Figure 1E.

The tagged loci were always observed as a single focus in newborn cells (Figure 1B and 2B). This is consistent with our growth conditions in which newborn cells contain a unique non-replicating copy of each of the two chromosomes. In dividing cells, two separate foci were observed for most of the analysed loci (Figures 1B and 2B). The only exceptions were *L5_I_*, *R5_I_* and *ter_I_*, whose foci were duplicated in about 50%, 40% and 10% of the dividing cells, respectively (Figure 1B). This is consistent with a previous report where sister copies of a locus situated at 40 kbp from *dif1* remained colocalised until the very end of septation [6].

The order of duplication of sister copies of each locus as a function of cell elongation followed the genetic map from *ori* to *ter* along the two arms of chromosome I and of chromosome II (Figures 1B and 2B). In addition, with the exception of the *dif1* proximal loci, the rate of focus duplication of any given locus was similar. The proportion of cells with two foci increased abruptly from 10% to above 80% within ≤0.6 µm of cell elongation. However, the difference in the timing of duplication of two consecutive loci was not strictly proportional to the genetic distance that separated them. For instance, sister *L1_I_* separation immediately followed sister *ori_I_* separation and occurred much earlier than sister *L2_I_* separation, despite *L1_I_* being closer to *L2_I_* than to *ori_I_*. This observation is consistent with a previous finding in *E. coli* suggesting that the time sister loci remain together after replication is variable [36]. In addition, it was sometimes not possible with the resolution of our experiments to differentiate the timing of duplication of loci that were too close on the genetic map, such as *ori_II_*, *R1_II_* and *R2_II_* or *R3_I_* and *R4_I_*.

Finally, using a strain with tagged *ori_II_* and *L3_I_*, it was observed that the separation of sister *L3_I_* occurred at smaller cell length than for sister *ori_II_* (Figure S9, ADV26). These results suggest that the separation of sister copies of the loci most proximal to the origin of replication on chromosome II occurred later than sister *ori_I_* separation (Figures 1B and 2B). This observation is in agreement with the delayed firing of the origin of replication of chromosome II compared to chromosome I replication [35].

### Longitudinal organisation of Chromosome I and II

For most loci and cell length categories, we observed cells with either two separated foci or a single focus. The proportion between these two types of cells varied as a function of cell elongation (Figures 1B and 2B). To simplify the representation of the data, we plotted the position of the foci that correspond to the dominant cell type in each cell length interval (Figures 1C and 2C). In addition, we plotted the median positions of the observed foci (filled circles), along with the 25^th^–75^th^ percentiles (error bars).

In newborn cells, the relative cell position of *ori_I_* and *ter_I_* was approximately 0.8 and 0.1 respectively, reflecting that *ori_I_* is positioned near the old pole and *ter_I_* close to the new pole. In agreement with previous reports the position of *ori_I_* in the overall cell population, i.e. irrespective of cell length, was closer to 0.9 [8], [20]. The distance between the position of *ori_I_* and the position of any locus on chromosome I correlated with the genetic distance, suggesting a longitudinal arrangement of the replichores within the cell (Figure 1C). To confirm this longitudinal arrangement we directly calculated the distance between *L3_I_*, which is located approximately in the middle of the left replichore, to additional chromosome I loci in cells that displayed a single focus for each of these two loci (Figure 1D). Consistent with a longitudinal arrangement *L3_I_* was observed as being closer to the right replichore loci than to *ori_I_* and *ter_I_* (Figure 1D). Furthermore, we directly calculated the distance between separated sister loci in individual cells (Figure 1E). The distance between sister copies of each locus linearly decreased from *ori_I_* to *ter_I_*, further confirming the longitudinal organisation of chromosome I (Figure 1E).

Two modes of segregation could be distinguished among chromosome I loci. The first mode of segregation was observed for loci located from *ori_I_* to *L4_I_* or *R4_I_*. In this mode, loci remained close to the position they occupied at cell birth until their duplication. As duplication occurred the greater the genetic distance between a locus and *ori_I_*, the longer it remained static at its home position. After duplication sister foci segregated to their new home positions in the next cell length interval, suggesting that segregation might be a transient event within the cell cycle. The distances travelled by two sister foci were not identical. The most unbalanced situation was observed for *ori_I_* where one copy remained nearly immobile whilst the second copy crossed the whole cell length. In contrast, the two *L3_I_* sister loci exhibited a symmetrical separation on either side of the midcell/future new pole. The second mode of segregation was characterized by the mobilization of the loci towards midcell before duplication. This mode of segregation applied to loci located in the terminus region of chromosome I, i.e. *R5_I_*, *L5_I_* and *ter_I_*. These loci migrated towards midcell (within 4 intervals) earlier than the duplication of loci located several hundreds of kb upstream (Figure 1B).

The *ori_II_* of chromosome II positioned near midcell (with a relative position of 0.55) and *dif2* near to the new pole (with a relative position of 0.28) in newborn cells. This is consistent with previous reports [6], [8]. The positioning and mobilisation of the *ori_II_* region depends on a partition system like *ori_I_* [15]. The positions of *R1_II_*, *R2_II_*, *R3_II_* and *L1_II_*, *L2_II_* were intermediate between those of *ori_II_* and *ter_II_*, suggesting that chromosome II occupied only the younger half of the cell. Despite the short distance separating *ori_II_* proximal loci (Figure 2B), the sequential *ori*_II_ to *ter*_II_ positioning of the seven chromosome II loci was observed, suggesting a longitudinal organisation (Figure 2C). Direct measurement of the distance between *ter*_II_ and *R1_I_*, *R2I*, *R3_I_* and *L2_II_* (Figure 2D) and of the distance between sister copies of all of the chromosomal loci (Figure 2E) confirmed the longitudinal organisation of chromosome II. Furthermore, the duplication of most loci occurred close to midcell (Figure 2C). This suggests that un-replicated chromosome II loci are pulled towards midcell by replication, which in turn pushes sister copies of replicated loci away from midcell and each other.

### Impact of *parS1* displacement on chromosome I organisation

We speculated that the displacement of *parS1* away from *oriCI* would modify the longitudinal arrangement of chromosome I. To test this hypothesis, tandem *parS1* sites were introduced at 300 kb, 490 kb and 650 kb on the left arm of chromosome I in cells devoid of their three natural *parS1* sites. Displacing the location of the Par-mediated anchoring zone on chromosome I did not affect the fitness or the morphology of the cells. The functionality of the displaced *parS1* sites was not affected as judged by the visualisation of at least one polar focus of Ypet-ParB1 in the majority of different cell populations (Figure S40).

**Figure 3:**
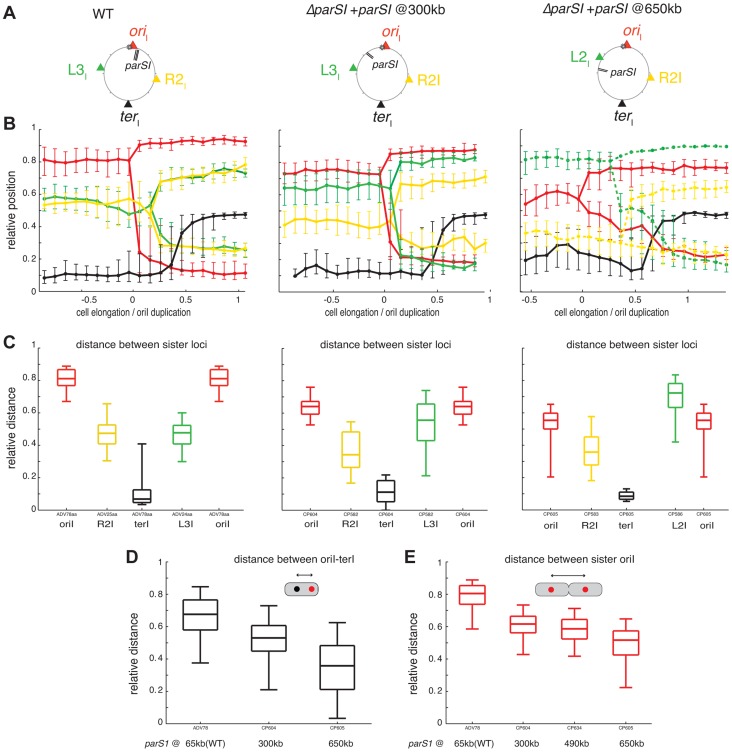
Reorganisation of chromosome I upon *parS1* displacement. (A) Circular map indicating the position of the different loci analysed in different genetic background (WT, *parS1*_300 kb_ and *parS1*_650 kb_ from left to right, respectively) and the colour code of the analysed loci. (B) Reconstitution of the segregation choreographies, as in Figure 1C. 2 µm of cell elongation were shown centred on the cell interval where at least 50% of the cells had duplicated *ori_I_*. For the WT choreography, ADV114 and ADV78 cell size distributions were aligned with the ADV24 cell sizes using the first cell size interval where 50% or more of cells contained duplicated *ori_I_*. For the *parS1*_300 kb_ choreography, CP604 and CP582 cell size distributions were aligned with the CP591 cell sizes using the first cell size interval where ≥50% of cells contained 2 foci of *ori_I_* and *L3_I_*, respectively. For the *parS1*_650 kb_ choreography, the cell sizes correspond to strain CP605 (*ori_I_* and *ter*_I_). CP583 and CP586 cell size distributions could not be realigned. The corresponding data was plotted with dashed lines (*L2_I_* and R2I). (C) Relative distances between any of the sister loci, as in Figure 1E. (D) Relative distance between any loci to *ter_I_* locus, as in Figure 1D. (E) Relative distance between *ori_I_* sister loci, in with displaced *parS1* sites, as in Figure 1E.

To assess the effect of *parS1* displacements on the global organization of chromosome I, the cellular positions of *ori_I_*, *R2_I_*, *L3_I_* and *ter_I_* were visualized. Displacing *parS1* sites on chromosome I had a dramatic effect on the positioning and segregation of *ori_I_*, *L3_I_* and *R2_I_* (Figure 3B). In contrast, the positioning and segregation of *ter_I_* was not significantly affected (Figure 3B). However, the displacement of *parS1* to the *L3_I_* location affected the polar positioning of *ter_I_* prior to its final recruitment to midcell.

The displacement of *parS1* sites to a location equidistant to *ori_I_* and *L3_I_*, caused the single *ori_I_* focus of small cells to shift towards midcell and the *ori_I_* sisters to be positioned closer to the quarter positions in long cells (Figure 3B, parS1@300kbp). This was confirmed by a decrease in the distance between *ori_I_* and *ter_I_* before *ori_I_* duplication (Figure 3D) and a decrease in the distance between *ori_I_* sisters after their separation (Figure 3C and 3E). The single *L3_I_* focus of small cells was shifted towards the old pole and *L3_I_* sisters were positioned further away from quarter positions in long cells (Figure 3B, parS1@300kbp). This was confirmed by the direct measurement of the distance between *L3_I_* sisters after their separation (Figure 3C). In addition, *R2_I_* positioning was shifted toward the new pole, as observed by a reduced distance between sister *R2_I_* loci (Figure 3C). As a consequence, *L3_I_* and *R2_I_* no longer co-localised (Figure 3B).

When the *parS1* sites were displaced to the *L3_I_* locus, the home position of *ori_I_* and *L2_I_* were switched. The *ori_I_* locus, which was now situated 700 kb away from the *parS1* sites, adopted the positioning of *L3_I_* locus in the wild-type context (Figure 3B, parS1@650kbp). This was confirmed by a decrease in the distance between *ori_I_* and *ter_I_* before *ori_I_* duplication (Figure 3D) and a decrease in the distance between *ori_I_* sisters after their separation (Figures 3C and 3E). Reciprocally, *L2_I_*, which was now located 170 kb from the *parS1* sites, exhibited a positioning similar to *ori_I_* in the wild-type cell (Figure 3B, parS1@650kbp). The distance between sister *L2_I_* also became larger than the *ori_I_* sister distance (Figure 3C). Finally, the shift of *R2_I_* positioning toward the new pole was exacerbated (Figure 3B and 3C).

**Figure 4:**
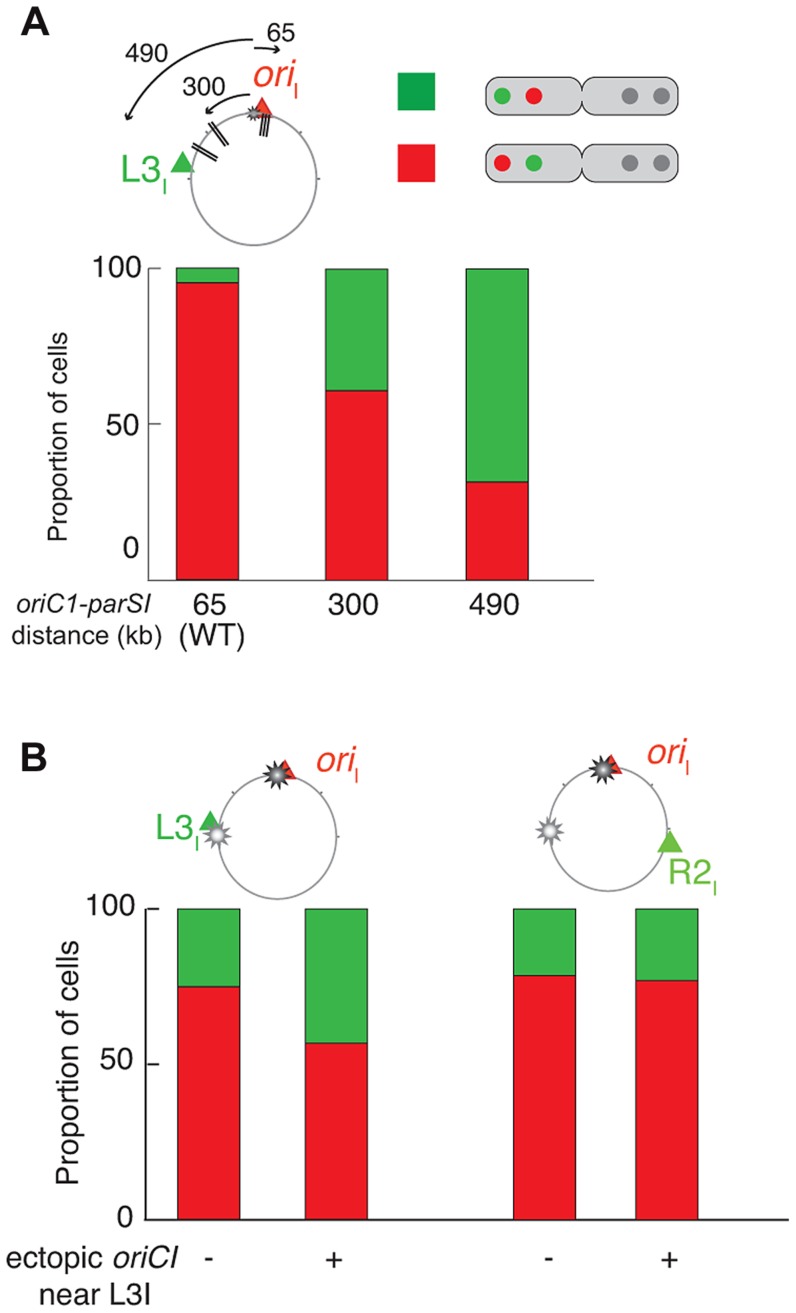
Increase of *L3_I_* polarity over *ori_I_* upon *parS1* or *oriC1* proximity to *L3_I_* locus. For each pole of dividing cells, the most polar locus between *ori_I_* and *L3_I_* was determined (A) in WT (ADV24), *parS1*_300 kb_ (CP591) and *parS1*_490 kb_ (CP634) and (B) in Δ*parS1* (CP568), Δ*parS1* + *oriC1*_651 kb_ (CP626), *parS1*_65 kb_ + *oriC1*_651 kb_ (CP633). This corresponds to approximately 100 cells. The red part of the stacked histogram represents the proportion of case with *ori_I_* more polar than *L3_I_*, whereas the green part represents the opposite.

To further demonstrate the extent of the chromosomal reorganisations, we took advantage of our double labelling systems to directly monitor the respective positions of *ori_I_* and *L3_I_* within long cells once they were duplicated and had reached their new home position. We determined the proportion of cells in which o*ri_I_* was more polar than *L3_I_* when the *parS1* sites were at their normal position (*parS1*_65 kb_, wild-type) or had been displaced by 300 kb (*parS1*_300 kb_) or 490 kb (*parS1*_490 kb_). This method allowed for chromosome rearrangements to be monitored at the single cell level. *ori_I_* was more polar than *L3_I_* in almost 100% of the cells in the wild type context (Figure 4A, *parS1*_65 kb_). The proportion of cells in which *ori_I_* was more polar than *L3_I_* decreased to 60% when *parS1* sites were equidistant between *ori_I_* and *L3_I_* (Figure 4A, *parS1*_300 kb_) and down to 30% when *parS1* sites were closer to *L3_I_* than to *ori_I_* (Figure 4A, *parS1*_490 kb_). The median distances between separated *ori_I_* sisters were very similar when *parS1* sites had been displaced to 300 kb or 490 kb (Figure 3E).

In conclusion, the displacement of *parS1* sites along the left arm of chromosome I led to the global rearrangement of chromosome I within the cell. These results suggest that the Par system of chromosome I not only mobilises and anchors the *parS1* sites at the poles but also directly contributes to the arrangement of the entire DNA molecule. Only the positioning of the terminus region escaped the influence of the Par system.

### Impact of *parS1* deletion on chromosome I organisation

**Figure 5:**
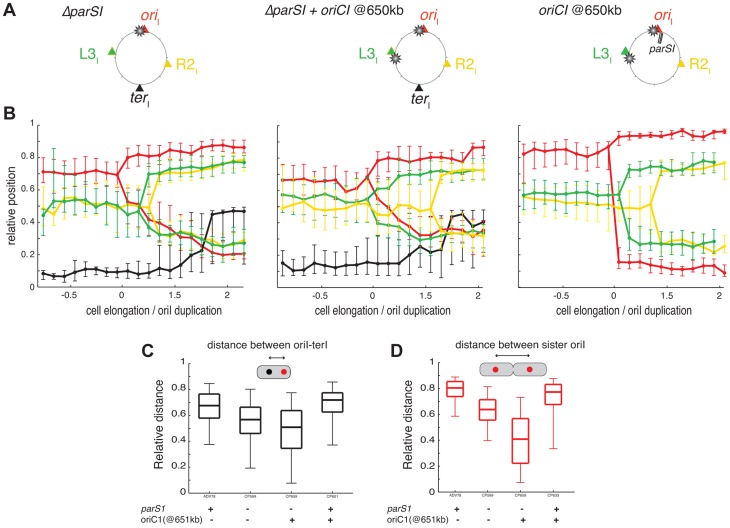
Disorganisation of chromosome I upon the ectopic *oriC1_651 kb_* addition. (A) Circular maps indicating the position of the different loci analysed in different genetic background from left to right, respectively : Δ*parS1*, Δ*parS1* + *oriC1*_651 kb_, *parS1*_65 kb_ + *oriC1*_651 kb_. (B) Reconstitution of the segregation choreographies of the loci tagged in the different genetic background, as in Figure 1C. For the Δ*parS1* choreography, the CP639 cell size distribution was aligned with the CP655 cell sizes using the cell size interval where ≥50% of cells contained two *R2_I_* foci; For the Δ*parS1* + *oriC1*_651 kb_ choreography, the cell sizes of CP626 and CP656 were aligned to the cell sizes of CP659 using the cell size interval where ≥50% of cells contained two *ori_I_* foci; For *parS1*_65 kb_ + *oriC1*_651 kb_ choreography, the cell sizes of ADV115 were realigned to the cell sizes of CP633 using the cell size interval where ≥50% of cells contained two *ori_I_* foci. (C) Relative distance between *ori_I_* and *ter_I_* loci, as in Figure 1D. (D) Relative distance between the *ori_I_* sister loci, as in Figure 1E.

Previous studies indicated that deletion of *parA1* resulted in the release of the *parS1* sites from the old pole with the relative position of ParB1 changing from 0.023 in a wild-type context to 0.2 [6], [8]. Despite this dramatic change, no effects on cell fitness or cell cycle parameters were reported, suggesting that chromosome I segregation was not affected by the disruption of its Par system. However, we speculated that the organisation and segregation choreography of chromosome I would be globally modified when Par*-*mediated mobilization and anchoring of *ori_I_* were lost. To this end, we followed the localisation of *ori_I_*, *R2_I_*, *L3_I_* and *ter_I_* loci in Δ*parS1* cells. The longitudinal arrangement of chromosome I was maintained during the whole cell cycle. *ori_I_* remained the closest loci to the old pole with a relative position of 0.7 in small cells and a relative *ori_I_* - *ter_I_* distance of 0.68 (Figures 5B and 5C). *R2_I_* and *L3_I_* loci remained co-localised during the whole cell cycle and were positioned around midcell until their duplication (Figure 5B). After duplication, *ori_I_* sisters remained more polar than *L3_I_* and *R2_I_* sisters, which still co-localised (Figure 5B). However, the relative distance between separated *ori_I_* sisters was smaller than in wild-type cells (Figure 5D). In addition, the increase in the 25^th^–75^th^ percentiles (error bars, Figure 5B) and the increase in the variability of the *ori_I_* - *ter_I_* distance (Figure 5C) suggest that the home positions of *ori_I_*, *L3_I_* and *R2_I_* before and after duplication were less stringently controlled. Time-lapse experiments confirmed the global behaviour of these three loci (data not shown). In contrast, the choreography and late duplication of *ter_I_* was not affected by the absence of the *parS1* sites. The positioning of *ori_II_*, *R2_II_* and *ter_II_* loci suggest that the chromosome II behaviour was not dramatically modified in Δ*parS1* cells (Figure S41). In conclusion, the impairment of the Par system of chromosome I slightly disorganized the positioning and the segregation of the bulk of chromosome I. However, chromosome I remained longitudinally organised and *ori_I_* remained more polar than the rest of chromosome I, suggesting the existence of additional chromosome I organizing and positioning mechanisms.

### Impact of an extra *oriC*1 at 651 kb on chromosome I organisation

We hypothesised that the replication program might be, at least in part, responsible for the maintenance of the longitudinal arrangement of chromosome I in the absence of *parS1* sites. To test this hypothesis, we introduced an extra origin of replication 651 kb from its normal position on the left replichore of chromosome I in cells lacking *parS1* sites. The fitness of cells harbouring the two origins was not affected. Replication profiling demonstrated that the ectopic origin was as efficient as the wild-type origin and that the two origins were used at each cell cycle (Figure S42). Correspondingly, the separation of *ori_I_* and *L3_I_* sisters became synchronous (Figure S42). The perturbation of the replication program further decreased the polarity of the *ori_I_* region in small cells (Figure 5B), which decreased the distance between single *ori_I_* and *ter_I_* foci (Figure 5C). The effect was more evident in long cells (Figure 5B) where the relative distance between separated *ori_I_* sisters decreased to half the distance of wild-type cells with a high level of variability (Figure 5D). The segregation of *ori_I_* sisters also became asymmetric with one of the two copies remaining relatively polar while the other adopted a more central position (Figure 5D). Finally, the *L3_I_* locus became slightly more polar and no longer co-localised with *R2_I_* (Figure 5B). In contrast, earlier replication of the *ter_I_* locus (Figure S42) did not modify its home position (Figure 5C). The position of the *R2_I_* locus was not significantly affected by the ectopic origin (Figure 5C). Then, we decided to directly monitor in each cells which of the two competing locus, *ori_I_* and *L3_I_*, was closer to the old pole. If the polar location of a given locus was only dictated by its proximity to the initiation of replication the proportion of cells in which *L3_I_* was closer to the old pole than *ori_I_* would reach 50%. Correspondingly, the polarity of *ori_I_* compared to *L3_I_* decreased from 80% in the parental cells (Δ*parS1*) to 55% in the cells harbouring an ectopic origin on the Left arm at 651 kb from *ori_I_* (Figure 4B). The polar location of *ori_I_* compared to *R2_I_* was unchanged by the addition of the ectopic origin, suggesting that the phenotype was not linked to a global disorganisation of the DNA within the cell (Figure 4B). Thus, the replication program itself contributes to the polar positioning of loci that are close to the origin of replication. However, this contribution is masked by the presence of a Par system (Figure 5B). The contribution of the replication program may be more evident in cells containing a unique but displaced *oriC1* site. However, the presence of an *oriC1* site within the origin region is essential for cell viability (Figure S43).

## Discussion

In this manuscript the use of fluorescent microscopy to follow the position of 19 loci within the genome of *V. cholerae* provides the first detailed characterization of the organization and dynamics of a multipartite bacterial genome.

### The two *V. cholerae* chromosomes are longitudinally arranged

**Figure 6:**
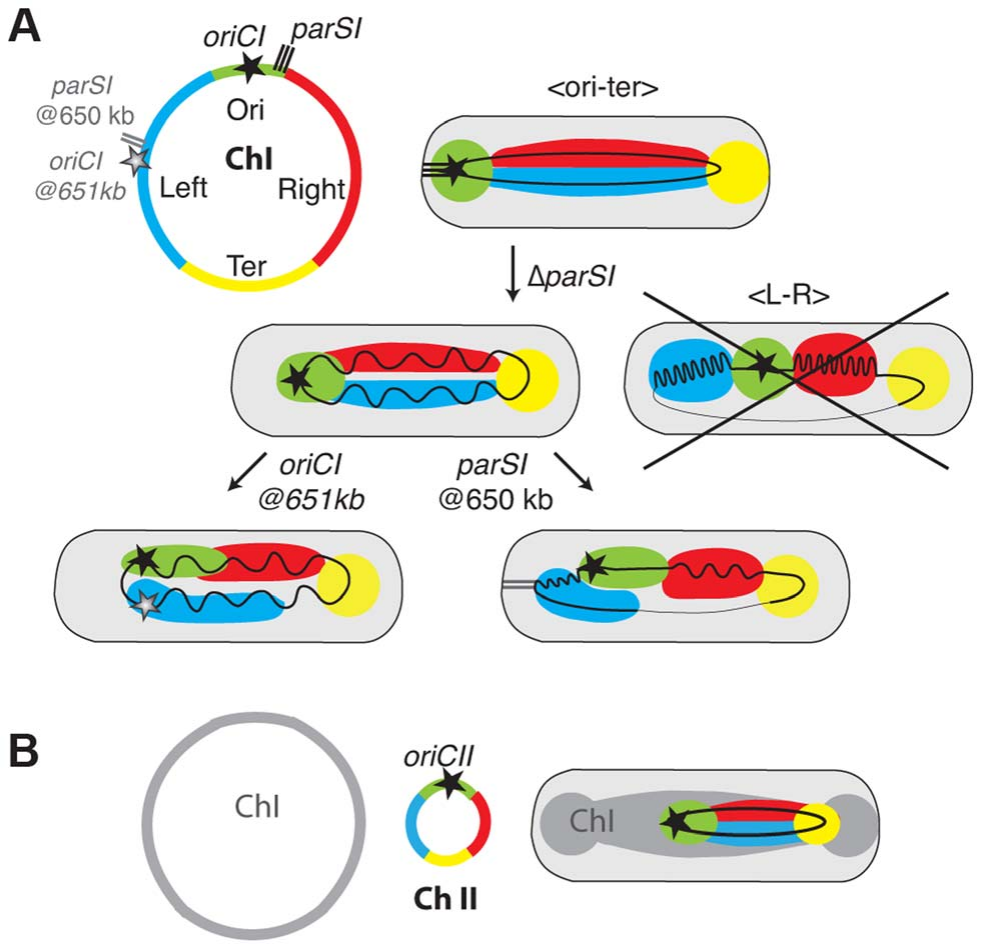
Models of chromosome I and II organisation and reorganisations by *parS1* and *oriC1* actions. (A) Chromosome I was divided in 4 regions Ori in green corresponding to the region proximal of the replication origin , Left in blue, Right in Red corresponding to the left and right replichores and Ter in Yellow corresponding to the *matS* sites containing region (circular map). In the WT context, the Ori and the Ter are confined to the old pole and new pole, respectively. The Left and Right are laying in between. In the Δ*parS1* context, the longitudinal organisation is maintained but the Ori is detached from the pole. The loss of *parS1* sites do not convert the organisation of chromosome I to a transversal type as in *E. coli* (crossed drawing). In the *parS1*_650 kb_ context, the Left becomes more polar than the Ori region. The Right is restricted toward the new pole where the Ter remains positioned. In the *oriC1*_651 kb_ context, the Left becomes as polar as the Ori but the chromosome is globally less organised. (B) The chromosome II was divided in 4 regions as for chromosome I. In the WT context, the Ori was confined to midcell. The Left and Right are extended from midcell to the new pole. The TerII is not closely tethered to the new pole as terI.

After division, the two replichores of chromosome I were arranged side by side from the old pole to the new pole (Figures 1 and 6A, WT). This longitudinal organization, with the origin positioned near the old pole and the terminus near the new pole in newborn cells, was expected for chromosome I due to the similarities in the positioning and dynamics of the origin and terminus regions with *C. crescentus* [30]. Previous reports suggested that the origin region of *V. cholerae* chromosome II was positioned at midcell in newborn cells and that, after replication, sister copies of it migrated to ¼ and ¾ positions [8]. This is similar to the positioning and dynamics of the *E. coli* chromosome, which suggested a transversal mode of organization for chromosome II. However, we demonstrate that the two replichores of chromosome II were arranged side by side with chromosome II only occupying the new half of the younger cells and the two sister chromatids the central part of the older cells (Figures 2 and 6B, WT). Thus, both *V. cholerae* chromosomes are longitudinally organized within the cell.

### Positioning and segregation dynamics of the terminus region

The terminus region of chromosome I behaved differently from the bulk of the chromosome and was recruited to midcell long before the time of sister foci duplication (Figure 1). In addition, terminus sister copies remained together until the very end of the cell cycle (Figure 1). Finally, whereas displacing *parS* sites led to the rotation of the whole chromosome in *C. crescentus* [30], the position of the terminus region of chromosome I was not affected by *parS1* displacements (Figure 3). These observations are likely due to the presence of a MatP/*matS* system in *V. cholerae* that is absent from *C. crescentus* [37], [38]. Our results also suggest that sister copies of the terminus region of chromosome II separated earlier than sister copies of the terminus region of chromosome I even though both regions harbour *matS* sites (Figure 2). The differential contribution of the MatP/*matS* macrodomain organization system to the segregation of the terminus regions of chromosome I and II is the subject of a another study [39].

### Longitudinal organisation is not entirely driven by partition systems

As for all other studied bacterial chromosomes with a longitudinal organization the position of the origin of replication region of each of the two *V. cholerae* chromosomes was driven by a ParABS partition system [40]. We speculate that the longitudinal organization of each chromosome is linked to the action of their partition machineries. Consistent with this, the displacement of *parS1* sites rearranged the bulk of chromosome I within the cell with the locus most proximal to the displaced *parS1* sites now occupying the polar edge of the chromosome (Figures 3, 4A and 6A, parS1@650kb). This is similar to the impact of *parS* sites on chromosome organisation in *C. crescentus* [30]. However, chromosome I remained longitudinally arranged in the absence of *parS1* sites suggesting the existence of additional factors involved in chromosomal organisation (Figures 5 and 6A, Δ*parS1*).

### Replication contributes to chromosome segregation in *V. cholerae*

In *C. crescentus* sister loci segregate independently of their timing of replication in an order roughly corresponding to their distance from *parS* sites [29], [41]. The results of this current study suggest that the segregation of chromosome I loci was no longer correlated to their genetic distance from *parS1* sites when these sites had been displaced (Figures 3 and S44), but instead followed the replication program. The efficient segregation of *V. cholerae* chromosome I lacking *parS1* sites suggests that *parS1*-polar mobilisation is not essential for the segregation process. The influence of the replication program on the organization and segregation of chromosome I was further assessed by inserting an ectopic origin of replication in the left replichore. The segregation and the organisation of the bulk of chromosome I was not altered if the partition machinery remained intact (Figure 5). However, when combined with the deletion of the *parS1* sites, the addition of an ectopic origin of replication affected the positioning of loci proximal to the two functional origins (Figures 5 and 4B). Taken together, these results suggest that the longitudinal organization of chromosome I was preserved by the replication program when its partition system was disrupted (Figures 5 and 6A, Δ*parS1*).

In *E. coli* and *B. subtilis*, modification of the replication program by the addition or the displacement of the replication origin does not influence the organisation of the bacterial chromosome [40], [42]. In particular, replication initiation from an origin located in the middle of the right replichore did not disrupt the transversal organisation of the *E. coli* chromosome [40]. However, it increased the proportion of cells in which the origin sisters were displaced to the outer edges of the nucleoid, i.e. towards the cell poles, suggesting that replication program could contribute to polar mobilisation in *E. coli*. Moreover, the *E. coli* chromosome lost its transversal organisation and adopted a longitudinal organisation in *mukB* mutant cells [43], [44]. Preliminary studies suggest that deletion of the *mukBEF* genes in *V. cholerae*, which does not confer any loss of cell viability or any aberrant cell morphology (data not shown), does not alter the positioning and segregation of *ori_I_* and *L3_I_* (Figure S24). Nevertheless, future work is needed to investigate the contribution of the MukBEF system to the organization and segregation of the two *V. cholerae* chromosomes.

In conclusion, this study demonstrates that the partition machinery and the replication program contribute to the polar mobilization of the origin regions in *V. cholerae.* We hypothesise that it is the genomic proximity of *parS* sites to the *oriC* site, which is a conserved feature of most bacterial chromosomes that serves to ensure the convergence of their polar positioning activities.

## Materials and Methods

### Plasmids and strains

Bacterial strains and plasmids used in this study are listed in Tables S1 and S2 respectively. All *V. cholerae* mutants were constructed by integration-excision or natural transformation (Protocols and details on the construction of each strain in Text S1). To this end, a derivative of the El Tor V. cholerae N16961 was rendered competent by the insertion of *hapR* by specific transposition [45]. Engineered strains were confirmed by PCR.

### Fluorescence microscopy

Cells were observed in Minimal Media to have only a single copy of each chromosome after division. Protocols for Microscopy are detailed in Text S1. The snapshot images were analysed using the Matlab-based software MicrobeTracker [45], [46], see details for the analysis in Supplementary Text S1.

## Supporting Information

Figure S1 Timelapses of ADV21 and ADV23. Acquisition was every 5 min and only timepoints of interest are shown. Green foci correspond to yGFP-ParB_pMT1_ and red foci to LacI-mCherry.
(EPS)

Figure S2 Cell size distribution of different strains studied. They are plotted against ADV24 (*ori_I_* and *L3_I_*) as reference.
(EPS)

Figure S3 Duplication frequency and reconstituted segregation choreography as a function of cell length in ADV20 strain (not realigned). Top panel: numbers of cells in each cell size interval (0.2 µm); Middle panel: proportion of cells with duplicated foci according to cell size for each tagged locus; Bottom panel: relative median, 1 quartile and 3 quartiles positions of each locus according to cell size elongation. The principle of cell orientation is indicated.
(EPS)

Figure S4 Duplication frequency and reconstituted segregation choreography as a function of cell length in ADV21 strain (not realigned). Top panel: numbers of cells in each cell size interval (0.2 µm); Middle panel: proportion of cells with duplicated foci according to cell size for each tagged locus; Bottom panel: relative median, 1 quartile and 3 quartiles positions of each locus according to cell size elongation. The principle of cell orientation is indicated.
(EPS)

Figure S5 Duplication frequency and reconstituted segregation choreography as a function of cell length in ADV22 strain (not realigned). Top panel: numbers of cells in each cell size interval (0.2 µm); Middle panel: proportion of cells with duplicated foci according to cell size for each tagged locus; Bottom panel: relative median, 1 quartile and 3 quartiles positions of each locus according to cell size elongation. The principle of cell orientation is indicated.
(EPS)

Figure S6 Duplication frequency and reconstituted segregation choreography as a function of cell length in ADV23 strain (not realigned). Top panel: numbers of cells in each cell size interval (0.2 µm); Middle panel: proportion of cells with duplicated foci according to cell size for each tagged locus; Bottom panel: relative median, 1 quartile and 3 quartiles positions of each locus according to cell size elongation. The principle of cell orientation is indicated.
(EPS)

Figure S7 Duplication frequency and reconstituted segregation choreography as a function of cell length in ADV24 strain (not realigned). Top panel: numbers of cells in each cell size interval (0.2 µm); Middle panel: proportion of cells with duplicated foci according to cell size for each tagged locus; Bottom panel: relative median, 1 quartile and 3 quartiles positions of each locus according to cell size elongation. The principle of cell orientation is indicated.
(EPS)

Figure S8 Duplication frequency and reconstituted segregation choreography as a function of cell length in ADV25 strain (not realigned). Top panel: numbers of cells in each cell size interval (0.2 µm); Middle panel: proportion of cells with duplicated foci according to cell size for each tagged locus; Bottom panel: relative median, 1 quartile and 3 quartiles positions of each locus according to cell size elongation. The principle of cell orientation is indicated.
(EPS)

Figure S9 Duplication frequency and reconstituted segregation choreography as a function of cell length in ADV26 strain (not realigned). Top panel: numbers of cells in each cell size interval (0.2 µm); Middle panel: proportion of cells with duplicated foci according to cell size for each tagged locus; Bottom panel: relative median, 1 quartile and 3 quartiles positions of each locus according to cell size elongation. The principle of cell orientation is indicated.
(EPS)

Figure S10 Duplication frequency and reconstituted segregation choreography as a function of cell length in ADV27 strain (not realigned). Top panel: numbers of cells in each cell size interval (0.2 µm); Middle panel: proportion of cells with duplicated foci according to cell size for each tagged locus; Bottom panel: relative median, 1 quartile and 3 quartiles positions of each locus according to cell size elongation. The principle of cell orientation is indicated.
(EPS)

Figure S11 Duplication frequency and reconstituted segregation choreography as a function of cell length in ADV30 strain (not realigned). Top panel: numbers of cells in each cell size interval (0.2 µm); Middle panel: proportion of cells with duplicated foci according to cell size for each tagged locus; Bottom panel: relative median, 1 quartile and 3 quartiles positions of each locus according to cell size elongation. The principle of cell orientation is indicated.
(EPS)

Figure S12 Duplication frequency and reconstituted segregation choreography as a function of cell length in ADV33 strain (not realigned). Top panel: numbers of cells in each cell size interval (0.2 µm); Middle panel: proportion of cells with duplicated foci according to cell size for each tagged locus; Bottom panel: relative median, 1 quartile and 3 quartiles positions of each locus according to cell size elongation. The principle of cell orientation is indicated.
(EPS)

Figure S13 Duplication frequency and reconstituted segregation choreography as a function of cell length in ADV39 strain (not realigned). Top panel: numbers of cells in each cell size interval (0.2 µm); Middle panel: proportion of cells with duplicated foci according to cell size for each tagged locus; Bottom panel: relative median, 1 quartile and 3 quartiles positions of each locus according to cell size elongation. The principle of cell orientation is indicated.
(EPS)

Figure S14 Duplication frequency and reconstituted segregation choreography as a function of cell length in ADV42 strain (not realigned). Top panel: numbers of cells in each cell size interval (0.2 µm); Middle panel: proportion of cells with duplicated foci according to cell size for each tagged locus; Bottom panel: relative median, 1 quartile and 3 quartiles positions of each locus according to cell size elongation. The principle of cell orientation is indicated.
(EPS)

Figure S15 Duplication frequency and reconstituted segregation choreography as a function of cell length in ADV50 strain (not realigned). Top panel: numbers of cells in each cell size interval (0.2 µm); Middle panel: proportion of cells with duplicated foci according to cell size for each tagged locus; Bottom panel: relative median, 1 quartile and 3 quartiles positions of each locus according to cell size elongation. The principle of cell orientation is indicated.
(EPS)

Figure S16 Duplication frequency and reconstituted segregation choreography as a function of cell length in ADV51 strain (not realigned). Top panel: numbers of cells in each cell size interval (0.2 µm); Middle panel: proportion of cells with duplicated foci according to cell size for each tagged locus; Bottom panel: relative median, 1 quartile and 3 quartiles positions of each locus according to cell size elongation. The principle of cell orientation is indicated.
(EPS)

Figure S17 Duplication frequency and reconstituted segregation choreography as a function of cell length in ADV78 strain (not realigned). Top panel: numbers of cells in each cell size interval (0.2 µm); Middle panel: proportion of cells with duplicated foci according to cell size for each tagged locus; Bottom panel: relative median, 1 quartile and 3 quartiles positions of each locus according to cell size elongation. The principle of cell orientation is indicated.
(EPS)

Figure S18 Duplication frequency and reconstituted segregation choreography as a function of cell length in ADV114 strain (not realigned). Top panel: numbers of cells in each cell size interval (0.2 µm); Middle panel: proportion of cells with duplicated foci according to cell size for each tagged locus; Bottom panel: relative median, 1 quartile and 3 quartiles positions of each locus according to cell size elongation. The principle of cell orientation is indicated.
(EPS)

Figure S19 Duplication frequency and reconstituted segregation choreography as a function of cell length in ADV115 strain (not realigned). Top panel: numbers of cells in each cell size interval (0.2 µm); Middle panel: proportion of cells with duplicated foci according to cell size for each tagged locus; Bottom panel: relative median, 1 quartile and 3 quartiles positions of each locus according to cell size elongation. The principle of cell orientation is indicated.
(EPS)

Figure S20 Duplication frequency and reconstituted segregation choreography as a function of cell length in ADV123 strain (not realigned). Top panel: numbers of cells in each cell size interval (0.2 µm); Middle panel: proportion of cells with duplicated foci according to cell size for each tagged locus; Bottom panel: relative median, 1 quartile and 3 quartiles positions of each locus according to cell size elongation. The principle of cell orientation is indicated.
(EPS)

Figure S21 Duplication frequency and reconstituted segregation choreography as a function of cell length in ADV128 strain (not realigned). Top panel: numbers of cells in each cell size interval (0.2 µm); Middle panel: proportion of cells with duplicated foci according to cell size for each tagged locus; Bottom panel: relative median, 1 quartile and 3 quartiles positions of each locus according to cell size elongation. The principle of cell orientation is indicated.
(EPS)

Figure S22 Duplication frequency and reconstituted segregation choreography as a function of cell length in ADV130 strain (not realigned). Top panel: numbers of cells in each cell size interval (0.2 µm); Middle panel: proportion of cells with duplicated foci according to cell size for each tagged locus; Bottom panel: relative median, 1 quartile and 3 quartiles positions of each locus according to cell size elongation. The principle of cell orientation is indicated.
(EPS)

Figure S23 Duplication frequency and reconstituted segregation choreography as a function of cell length in ADV131 strain (not realigned). Top panel: numbers of cells in each cell size interval (0.2 µm); Middle panel: proportion of cells with duplicated foci according to cell size for each tagged locus; Bottom panel: relative median, 1 quartile and 3 quartiles positions of each locus according to cell size elongation. The principle of cell orientation is indicated.
(EPS)

Figure S24 Duplication frequency and reconstituted segregation choreography as a function of cell length in CP454 strain (not realigned). Top panel: numbers of cells in each cell size interval (0.2 µm); Middle panel: proportion of cells with duplicated foci according to cell size for each tagged locus; Bottom panel: relative median, 1 quartile and 3 quartiles positions of each locus according to cell size elongation. The principle of cell orientation is indicated.
(EPS)

Figure S25 Duplication frequency and reconstituted segregation choreography as a function of cell length in CP560 strain (not realigned). Top panel: numbers of cells in each cell size interval (0.2 µm); Middle panel: proportion of cells with duplicated foci according to cell size for each tagged locus; Bottom panel: relative median, 1 quartile and 3 quartiles positions of each locus according to cell size elongation. The principle of cell orientation is indicated.
(EPS)

Figure S26 Duplication frequency and reconstituted segregation choreography as a function of cell length in CP568 strain (not realigned). Top panel: numbers of cells in each cell size interval (0.2 µm); Middle panel: proportion of cells with duplicated foci according to cell size for each tagged locus; Bottom panel: relative median, 1 quartile and 3 quartiles positions of each locus according to cell size elongation. The principle of cell orientation is indicated.
(EPS)

Figure S27 Duplication frequency and reconstituted segregation choreography as a function of cell length in CP582 strain (not realigned). Top panel: numbers of cells in each cell size interval (0.2 µm); Middle panel: proportion of cells with duplicated foci according to cell size for each tagged locus; Bottom panel: relative median, 1 quartile and 3 quartiles positions of each locus according to cell size elongation. The principle of cell orientation is indicated.
(EPS)

Figure S28 Duplication frequency and reconstituted segregation choreography as a function of cell length in CP591 strain (not realigned). Top panel: numbers of cells in each cell size interval (0.2 µm); Middle panel: proportion of cells with duplicated foci according to cell size for each tagged locus; Bottom panel: relative median, 1 quartile and 3 quartiles positions of each locus according to cell size elongation. The principle of cell orientation is indicated.
(EPS)

Figure S29 Duplication frequency and reconstituted segregation choreography as a function of cell length in CP599 strain (not realigned). Top panel: numbers of cells in each cell size interval (0.2 µm); Middle panel: proportion of cells with duplicated foci according to cell size for each tagged locus; Bottom panel: relative median, 1 quartile and 3 quartiles positions of each locus according to cell size elongation. The principle of cell orientation is indicated.
(EPS)

Figure S30 Duplication frequency and reconstituted segregation choreography as a function of cell length in CP604 strain (not realigned). Top panel: numbers of cells in each cell size interval (0.2 µm); Middle panel: proportion of cells with duplicated foci according to cell size for each tagged locus; Bottom panel: relative median, 1 quartile and 3 quartiles positions of each locus according to cell size elongation. The principle of cell orientation is indicated.
(EPS)

Figure S31 Duplication frequency and reconstituted segregation choreography as a function of cell length in CP605 strain (not realigned). Top panel: numbers of cells in each cell size interval (0.2 µm); Middle panel: proportion of cells with duplicated foci according to cell size for each tagged locus; Bottom panel: relative median, 1 quartile and 3 quartiles positions of each locus according to cell size elongation. The principle of cell orientation is indicated.
(EPS)

Figure S32 Duplication frequency and reconstituted segregation choreography as a function of cell length in CP626 strain (not realigned). Top panel: numbers of cells in each cell size interval (0.2 µm); Middle panel: proportion of cells with duplicated foci according to cell size for each tagged locus; Bottom panel: relative median, 1 quartile and 3 quartiles positions of each locus according to cell size elongation. The principle of cell orientation is indicated.
(EPS)

Figure S33 Duplication frequency and reconstituted segregation choreography as a function of cell length in CP633 strain (not realigned). Top panel: numbers of cells in each cell size interval (0.2 µm); Middle panel: proportion of cells with duplicated foci according to cell size for each tagged locus; Bottom panel: relative median, 1 quartile and 3 quartiles positions of each locus according to cell size elongation. The principle of cell orientation is indicated.
(EPS)

Figure S34 Duplication frequency and reconstituted segregation choreography as a function of cell length in CP634 strain (not realigned). Top panel: numbers of cells in each cell size interval (0.2 µm); Middle panel: proportion of cells with duplicated foci according to cell size for each tagged locus; Bottom panel: relative median, 1 quartile and 3 quartiles positions of each locus according to cell size elongation. The principle of cell orientation is indicated.
(EPS)

Figure S35 Duplication frequency and reconstituted segregation choreography as a function of cell length in CP655 strain (not realigned). Top panel: numbers of cells in each cell size interval (0.2 µm); Middle panel: proportion of cells with duplicated foci according to cell size for each tagged locus; Bottom panel: relative median, 1 quartile and 3 quartiles positions of each locus according to cell size elongation. The principle of cell orientation is indicated.
(EPS)

Figure S36 Duplication frequency and reconstituted segregation choreography as a function of cell length in CP656 strain (not realigned). Top panel: numbers of cells in each cell size interval (0.2 µm); Middle panel: proportion of cells with duplicated foci according to cell size for each tagged locus; Bottom panel: relative median, 1 quartile and 3 quartiles positions of each locus according to cell size elongation. The principle of cell orientation is indicated.
(EPS)

Figure S37 Duplication frequency and reconstituted segregation choreography as a function of cell length in CP659 strain (not realigned). Top panel: numbers of cells in each cell size interval (0.2 µm); Middle panel: proportion of cells with duplicated foci according to cell size for each tagged locus; Bottom panel: relative median, 1 quartile and 3 quartiles positions of each locus according to cell size elongation. The principle of cell orientation is indicated.
(EPS)

Figure S38 Duplication frequency and reconstituted segregation choreography as a function of cell length in CP708 strain (not realigned). Top panel: numbers of cells in each cell size interval (0.2 µm); Middle panel: proportion of cells with duplicated foci according to cell size for each tagged locus; Bottom panel: relative median, 1 quartile and 3 quartiles positions of each locus according to cell size elongation. The principle of cell orientation is indicated.
(EPS)

Figure S39 Duplication frequency and reconstituted segregation choreography as a function of cell length in EPV213 strain (not realigned). Top panel: numbers of cells in each cell size interval (0.2 µm); Middle panel: proportion of cells with duplicated foci according to cell size for each tagged locus; Bottom panel: relative median, 1 quartile and 3 quartiles positions of each locus according to cell size elongation. The principle of cell orientation is indicated.
(EPS)

Figure S40 Localisation of Ypet-ParB1 in WT, Δ*parS1*, *parS1_300 kb_* , *parS1_650 kb_*. Overlay of Phase contrast (red) and GFP images (green) showing representative cells of each strains grown in M9 Fructose Thiamine medium.
(EPS)

Figure S41 Chromosome II positioning is not dramatically modified in Δ*parS1* cells. (A) Circular map indicating the position of the different loci analysed in the different genetic backgrounds: WT (left) and **Δ** *parS1* (right). (B) Reconstitution of the segregation choreographies, as in Figure 1C. For the WT choreography, strain cell sizes were realigned as in Figure 2. For the **Δ** *parS1* choreography, the cell sizes of ADV128 were aligned to the cell size of ADV27 using the first cell size interval where ≥50% of cells contained 2 foci of *ori_II_* and *R1_II_*, respectively. (C) Relative distance between *R1_II_* and *ter_II_* loci measured in the cells containing only one focus of each, as in Figure 1D. The positions of these 4 loci follow the order of the genetic map, from midcell (oriII) to the new pole (terII). Note, that the variability of the *terII* locus positioning was increased in Δ*parS1* cells.
(EPS)

Figure S42 Functionality of the ectopic *oriC1_651 kb_* origin of replication. (A) Marker Frequency Analysis of chromosome I in exponentially growing WT (ADV24) and *oriC1_651 kb_* (CP626) cells. The presence of sharp peaks of equivalent heights at the two origins of strain CP626 indicates that they simultaneously fired at each round of replication. (B) Proportion of the different cell types in WT (ADV24) and in *oriC1_651 kb_* (CP626).
(EPS)

Figure S43 Presence of *oriC1* site is essential within the origin region. After introducing an ectopic *oriC1* using natural transformation with pPOS228, we attempted to delete the original Origin of replication of the chromosome I. Two strategies were performed: a direct deletion by replacing the *oriC1* with a Rif resistance gene (pAD43), or an indirect deletion, by first replacing *oriC1* by an *oriC1* flanked by FRT sites (pAD44) and secondly deleting it by inducing a Flipase protein (pFLP2). When the second *oriC1* was introduced in the middle of the replichore (near *L3_I_* locus), we were unable to delete the original *oriC1*, even thought it was possible to replace it by a FRT-*oriC1*-FRT. It was however possible to delete this Origin when the second *oriC1* was at only 50 kb from it.
(EPS)

Figure S44 Proportion of the different cell types in WT (ADV24) and in *parS1_490 kb_* (CP634).
(EPS)

Table S1 Strains list.
(DOCX)

Table S2 Plasmids list.
(DOCX)

Text S1 Supplementary Material and Methods.
(DOCX)

## References

[1] Kuempel PL, Henson JM, Dircks L, Tecklenburg M, Lim DF (1991) dif, a recA-independent recombination site in the terminus region of the chromosome of Escherichia coli. New Biol 3: 799–811. 1657123

[2] Possoz C, Junier I, Espeli O (2012) Bacterial chromosome segregation. Front Biosci 17: 1020–1034. 10.2741/3971 22201788

[3] Vallet-Gely I, Boccard F (2013) Chromosomal organization and segregation in Pseudomonas aeruginosa. PLoS Genet 9: e1003492. 23658532 10.1371/journal.pgen.1003492 PMC3642087

[4] Harms A, Treuner-Lange A, Schumacher D, Sogaard-Andersen L Tracking of Chromosome and Replisome Dynamics in Myxococcus xanthus Reveals a Novel Chromosome Arrangement. PLoS Genet 9: e1003802. 24068967 10.1371/journal.pgen.1003802 PMC3778016

[5] Viollier PH, Shapiro L (2004) Spatial complexity of mechanisms controlling a bacterial cell cycle. Curr Opin Microbiol 7: 572–578. 15556028 10.1016/j.mib.2004.10.005

[6] Srivastava P, Fekete RA, Chattoraj DK (2006) Segregation of the replication terminus of the two Vibrio cholerae chromosomes. J Bacteriol 188: 1060–1070. 16428410 10.1128/JB.188.3.1060-1070.2006 PMC1347332

[7] Fiebig A, Keren K, Theriot JA (2006) Fine-scale time-lapse analysis of the biphasic, dynamic behaviour of the two Vibrio cholerae chromosomes. Mol Microbiol 60: 1164–1178. 16689793 10.1111/j.1365-2958.2006.05175.x PMC2779472

[8] Fogel MA, Waldor MK (2006) A dynamic, mitotic-like mechanism for bacterial chromosome segregation. Genes Dev 20: 3269–3282. 17158745 10.1101/gad.1496506 PMC1686604

[9] Livny J, Yamaichi Y, Waldor MK (2007) Distribution of centromere-like parS sites in bacteria: insights from comparative genomics. J Bacteriol 189: 8693–8703. 17905987 10.1128/JB.01239-07 PMC2168934

[10] Vecchiarelli AG, Mizuuchi K, Funnell BE Surfing biological surfaces: exploiting the nucleoid for partition and transport in bacteria. Mol Microbiol 86: 513–523. 22934804 10.1111/mmi.12017 PMC3481007

[11] Salje J, Gayathri P, Lowe J (2010) The ParMRC system: molecular mechanisms of plasmid segregation by actin-like filaments. Nat Rev Microbiol 8: 683–692. 20844556 10.1038/nrmicro2425

[12] Ogura T, Hiraga S (1983) Partition mechanism of F plasmid: two plasmid gene-encoded products and a cis-acting region are involved in partition. Cell 32: 351–360. 6297791 10.1016/0092-8674(83)90454-3

[13] Martin KA, Friedman SA, Austin SJ (1987) Partition site of the P1 plasmid. Proc Natl Acad Sci U S A 84: 8544–8547. 3317415 10.1073/pnas.84.23.8544 PMC299581

[14] Thanbichler M, Shapiro L (2006) MipZ, a spatial regulator coordinating chromosome segregation with cell division in Caulobacter. Cell 126: 147–162. 16839883 10.1016/j.cell.2006.05.038

[15] Yamaichi Y, Fogel MA, Waldor MK (2007) par genes and the pathology of chromosome loss in Vibrio cholerae. Proc Natl Acad Sci U S A 104: 630–635. 17197419 10.1073/pnas.0608341104 PMC1760642

[16] Lasocki K, Bartosik AA, Mierzejewska J, Thomas CM, Jagura-Burdzy G (2007) Deletion of the parA (soj) homologue in Pseudomonas aeruginosa causes ParB instability and affects growth rate, chromosome segregation, and motility. J Bacteriol 189: 5762–5772. 17545287 10.1128/JB.00371-07 PMC1951838

[17] Ireton K, Gunther NWt, Grossman AD (1994) spo0J is required for normal chromosome segregation as well as the initiation of sporulation in Bacillus subtilis. J Bacteriol 176: 5320–5329. 8071208 10.1128/jb.176.17.5320-5329.1994 PMC196717

[18] Yamaichi Y, Fogel MA, McLeod SM, Hui MP, Waldor MK (2007) Distinct centromere-like parS sites on the two chromosomes of Vibrio spp. J Bacteriol 189: 5314–5324. 17496089 10.1128/JB.00416-07 PMC1951861

[19] Murray H, Errington J (2008) Dynamic control of the DNA replication initiation protein DnaA by Soj/ParA. Cell 135: 74–84. 18854156 10.1016/j.cell.2008.07.044

[20] Kadoya R, Baek JH, Sarker A, Chattoraj DK (2011) Participation of chromosome segregation protein ParAI of Vibrio cholerae in chromosome replication. J Bacteriol 193: 1504–1514. 21257772 10.1128/JB.01067-10 PMC3067663

[21] Gruber S, Errington J (2009) Recruitment of condensin to replication origin regions by ParB/SpoOJ promotes chromosome segregation in B. subtilis. Cell 137: 685–696. 19450516 10.1016/j.cell.2009.02.035

[22] Sullivan NL, Marquis KA, Rudner DZ (2009) Recruitment of SMC by ParB-parS organizes the origin region and promotes efficient chromosome segregation. Cell 137: 697–707. 19450517 10.1016/j.cell.2009.04.044 PMC2892783

[23] Minnen A, Attaiech L, Thon M, Gruber S, Veening JW (2011) SMC is recruited to oriC by ParB and promotes chromosome segregation in Streptococcus pneumoniae. Mol Microbiol 81: 676–688. 21651626 10.1111/j.1365-2958.2011.07722.x

[24] Ptacin JL, Lee SF, Garner EC, Toro E, Eckart M, et al (2010) A spindle-like apparatus guides bacterial chromosome segregation. Nat Cell Biol 12: 791–798. 20657594 10.1038/ncb2083 PMC3205914

[25] Schofield WB, Lim HC, Jacobs-Wagner C (2010) Cell cycle coordination and regulation of bacterial chromosome segregation dynamics by polarly localized proteins. EMBO J 29: 3068–3081. 20802464 10.1038/emboj.2010.207 PMC2944072

[26] Shebelut CW, Guberman JM, van Teeffelen S, Yakhnina AA, Gitai Z (2010) Caulobacter chromosome segregation is an ordered multistep process. Proc Natl Acad Sci U S A 107: 14194–14198. 20660743 10.1073/pnas.1005274107 PMC2922572

[27] Yamaichi Y, Bruckner R, Ringgaard S, Moll A, Cameron DE, et al (2012) A multidomain hub anchors the chromosome segregation and chemotactic machinery to the bacterial pole. Genes Dev 26: 2348–2360. 23070816 10.1101/gad.199869.112 PMC3475806

[28] Laloux G, Jacobs-Wagner C (2013) Spatiotemporal control of PopZ localization through cell cycle-coupled multimerization. J Cell Biol 201: 827–841. 23751494 10.1083/jcb.201303036 PMC3678156

[29] Viollier PH, Thanbichler M, McGrath PT, West L, Meewan M, et al (2004) Rapid and sequential movement of individual chromosomal loci to specific subcellular locations during bacterial DNA replication. Proc Natl Acad Sci U S A 101: 9257–9262. 15178755 10.1073/pnas.0402606101 PMC438963

[30] Umbarger MA, Toro E, Wright MA, Porreca GJ, Bau D, et al (2011) The three-dimensional architecture of a bacterial genome and its alteration by genetic perturbation. Mol Cell 44: 252–264. 22017872 10.1016/j.molcel.2011.09.010 PMC3874842

[31] Fogel MA, Waldor MK (2005) Distinct segregation dynamics of the two Vibrio cholerae chromosomes. Mol Microbiol 55: 125–136. 15612922 10.1111/j.1365-2958.2004.04379.x

[32] Kahng LS, Shapiro L (2003) Polar localization of replicon origins in the multipartite genomes of Agrobacterium tumefaciens and Sinorhizobium meliloti. J Bacteriol 185: 3384–3391. 12754237 10.1128/JB.185.11.3384-3391.2003 PMC155372

[33] Wang X, Liu X, Possoz C, Sherratt DJ (2006) The two Escherichia coli chromosome arms locate to separate cell halves. Genes Dev 20: 1727–1731. 16818605 10.1101/gad.388406 PMC1522069

[34] Nielsen HJ, Ottesen JR, Youngren B, Austin SJ, Hansen FG (2006) The Escherichia coli chromosome is organized with the left and right chromosome arms in separate cell halves. Mol Microbiol 62: 331–338. 17020576 10.1111/j.1365-2958.2006.05346.x

[35] Rasmussen T, Jensen RB, Skovgaard O (2007) The two chromosomes of Vibrio cholerae are initiated at different time points in the cell cycle. EMBO J 26: 3124–3131. 17557077 10.1038/sj.emboj.7601747 PMC1914095

[36] Joshi MC, Bourniquel A, Fisher J, Ho BT, Magnan D, et al (2011) Escherichia coli sister chromosome separation includes an abrupt global transition with concomitant release of late-splitting intersister snaps. Proc Natl Acad Sci U S A 108: 2765–2770. 21282646 10.1073/pnas.1019593108 PMC3041144

[37] Brezellec P, Hoebeke M, Hiet MS, Pasek S, Ferat JL (2006) DomainSieve: a protein domain-based screen that led to the identification of dam-associated genes with potential link to DNA maintenance. Bioinformatics 22: 1935–1941. 16787973 10.1093/bioinformatics/btl336

[38] Mercier R, Petit MA, Schbath S, Robin S, El Karoui M, et al (2008) The MatP/matS site-specific system organizes the terminus region of the E. coli chromosome into a macrodomain. Cell 135: 475–485. 18984159 10.1016/j.cell.2008.08.031

[39] Demarre G, Galli E, Muresan L, Paly E, David A, et al (2014) Differential management of the replication terminus regions of the two Vibrio cholerae chromosomes during cell division. PLoS Genet [In Press]. 10.1371/journal.pgen.1004557 PMC4177673 25255436

[40] Wang X, Lesterlin C, Reyes-Lamothe R, Ball G, Sherratt DJ (2011) Replication and segregation of an Escherichia coli chromosome with two replication origins. Proc Natl Acad Sci U S A 108: E243–250. 21670292 10.1073/pnas.1100874108 PMC3127894

[41] Toro E, Hong SH, McAdams HH, Shapiro L (2008) Caulobacter requires a dedicated mechanism to initiate chromosome segregation. Proc Natl Acad Sci U S A 105: 15435–15440. 18824683 10.1073/pnas.0807448105 PMC2563096

[42] Berkmen MB, Grossman AD (2007) Subcellular positioning of the origin region of the Bacillus subtilis chromosome is independent of sequences within oriC, the site of replication initiation, and the replication initiator DnaA. Mol Microbiol 63: 150–165. 17140409 10.1111/j.1365-2958.2006.05505.x

[43] Danilova O, Reyes-Lamothe R, Pinskaya M, Sherratt D, Possoz C (2007) MukB colocalizes with the oriC region and is required for organization of the two Escherichia coli chromosome arms into separate cell halves. Mol Microbiol 65: 1485–1492. 17824928 10.1111/j.1365-2958.2007.05881.x PMC2169520

[44] Badrinarayanan A, Lesterlin C, Reyes-Lamothe R, Sherratt D (2012) The Escherichia coli SMC complex, MukBEF, shapes nucleoid organization independently of DNA replication. J Bacteriol 194: 4669–4676. 22753058 10.1128/JB.00957-12 PMC3415497

[45] Marvig RL, Blokesch M Natural transformation of Vibrio cholerae as a tool–optimizing the procedure. BMC Microbiol 10: 155. 20509862 10.1186/1471-2180-10-155 PMC2890613

[46] Sliusarenko O, Heinritz J, Emonet T, Jacobs-Wagner C High-throughput, subpixel precision analysis of bacterial morphogenesis and intracellular spatio-temporal dynamics. Mol Microbiol 80: 612–627. 21414037 10.1111/j.1365-2958.2011.07579.x PMC3090749

