# Supplementary material for: The Two Cis-Acting Sites, parS1 and oriC1, Contribute to the Longitudinal Organisation of Vibrio cholerae Chromosome I
Source: PLoS Genet. 2014 Jul 10;10(7):e1004448. doi: 10.1371/journal.pgen.1004448 (PMC4091711; doi:10.1371/journal.pgen.1004448)
Supplement: Table S1 — Strains list. (DOCX) [file pgen.1004448.s045.docx]

Table S1

*Vibrio cholerae* strains

| **Chromosome I** | | All strains derived from ADV7 : N16961 hapR+, lacZ ::(LacI-mCherry , yGFP-parBT1) | | | | | | |  |
| --- | --- | --- | --- | --- | --- | --- | --- | --- | --- |
| Locus position  Genbank N16961  (Mbp) | Labelling  system | wt | ΔparS1 | ΔparS1+2parS1  @300kb | ΔparS1+ 2parS1  @490kb | ΔparS1+ 2parS1@650kb | Ori@  651kb | ΔparS1+ ori651kb | ΔmukBEF |
| Ori1 (0.053) | parS_PMT1_  lacO | ADV78, ADV114  ADV24, ADV123 | CP599,CP655  CP568 | CP604 | CP634 | CP605 | ADV115  CP633 | CP659, CP656  CP626 | CP454 |
| R1I (2.502) | lacO | ADV23 |  |  |  |  |  |  |  |
| R2I (2.237) | lacO | ADV25, ADV114 | CP639, CP655 | CP582 |  | CP583 | ADV115 | CP656 |  |
| R3I (2.088) | lacO | ADV21 |  |  |  |  |  |  |  |
| R4I (1.898) | lacO | ADV33 |  |  |  |  |  |  |  |
| R5I (1.686) | lacO | ADV51 |  |  |  |  |  |  |  |
| TerI (1.564) | lacO  tetO | ADV78, ADV42 | CP599,  CP639 | CP604 |  | CP605 |  | CP659 |  |
| L1I (0.426) | lacO | ADV22 |  |  |  |  |  |  |  |
| L2I (0.612) | lacO | ADV20 | ADV39 |  |  | CP586 |  |  |  |
| L3I (0.774) | parS_PMT1_ | ADV20, ADV21, ADV22, ADV24, ADV24, ADV25, ADV26, ADV30, ADV33, ADV42, ADV50, ADV51 | CP568,CP639 | CP582 | CP634 |  | CP633 | CP626 | CP454 |
| L4I (1.023) | parS_PMT1_ | EPV213 |  |  |  |  |  |  |  |
| L5I (1.461) | lacO | ADV50 |  |  |  |  |  |  |  |
| **Chromosome II** | |  |  |  |  |  |  |  |  |
| OriII (1.048 ) | lacO | ADV26 | ADV27 |  |  |  |  |  |  |
| R1II (0.064) | parS_PMT1_ | CP708 | ADV128 |  |  |  |  |  |  |
| R2II (0.184 ) | parS_PMT1_ | ADV131 |  |  |  |  |  |  |  |
| R3II (0.312) | parST1 | ADV30 | ADV27 |  |  |  |  |  |  |
| TerII (0.498) | lacO | ADV30, ADV130  ADV131, CP708 | ADV128 |  |  |  |  |  |  |
| L1II (0.820) | parS_PMT1_ | ADV123 |  |  |  |  |  |  |  |
| LII2 (0.659) | parS_PMT1_ | ADV130 |  |  |  |  |  |  |  |

For each strain of the table, its genotype is indicated in the corresponding column head and one of the locus labelled and the system used to label it are indicated in the corresponding line head. As a strain can present several labelled loci, a strain is entered in the table several times. For each strain, the orientation of the cells is given by the colour code of the strain name: red correspond to a locus (of corresponding line head) being oriented preferentially toward the old pole, blue correspond to a locus being oriented preferentially toward the new pole and black correspond to a locus which cannot be oriented.
