# Supplementary material for: The Two Cis-Acting Sites, parS1 and oriC1, Contribute to the Longitudinal Organisation of Vibrio cholerae Chromosome I
Source: PLoS Genet. 2014 Jul 10;10(7):e1004448. doi: 10.1371/journal.pgen.1004448 (PMC4091711; doi:10.1371/journal.pgen.1004448)
Supplement: Table S2 — Plasmids list. (DOCX) [file pgen.1004448.s046.docx]

Table S2

Table of Plasmids

| Name | Genotype | Reference |
| --- | --- | --- |
| P GD162 | integration-excision vector ; *sacB* ; ori R6K ;  Tet’-zeo-‘Tet cassette ; CmR, ZeoR | This study |
| pAD19 | integration-excision vector ; *sacB* ; ori R6K ; YGFP-ParBT1 and LacI-mcherry between Up- and Down-stream regions of *V.cholerae* *LacZ* gene ; CmR | This study |
| pAD20 | integration-excision vector ; *sacB* ; ori R6K ; LacO array + KanR between TetR homologies ; CmR, Kan | This study |
| pAD22 | integration-excision vector ; *sacB* ; Tet-Zeo-Tet cassette between homologies around LII ; CmR, ZeoR | This study |
| pAD23 | integration-excision vector ; *sacB* ; ori R6K ; Upstream of dif1- **dif1** – Tet’-zeo-‘Tet – Downstream of dif1 ; CmR, ZeoR, AmpR | This study |
| pAD24 | integration-excision vector ; *sacB* ; ori R6K ; Upstream of dif1 - **no dif1** – Tet’-zeo-‘Tet – Downstream of dif1 ; CmR, ZeoR, AmpR | This study |
| pAD25 | integration-excision vector ; *sacB* ; ori R6K ; Tet’-zeo-‘tet cassette between Up and Down homology zones on the right of dif1 at 1,68 Mb (1685991 bp) ; genes clockwise ; CmR, ZeoR | This study |
| pAD30b | pUC vector ; Upstream of dif2 - **dif2** – Tet’-zeo-‘Tet – Downstream of dif2 ; ZeoR, AmpR | This study |
| pAD31a | pUC vector ; Upstream of dif2 - **no dif2** – Tet’-zeo-‘Tet – Downstream of dif2 ; ZeoR, AmpR | This study |
| pAD39 | integration excision vector ; *sacB* ; ori R6K ;  Tet’-parST1- FRT-CmR-FRT-‘Tet ; CmR | This study |
| pPOS169 | integration excision vector ; *sacB* ; ori R6K ;  ΔparSI :: Spec | This study |
| pPOS184 | integration-excision vector ; *sacB* ; ori R6K ; *parSI*-FRT-Rif-FRT-*parSI* between TetR homologies ; CmR, Rif | This study |
| pPOS185 | integration-excision vector ; *sacB* ; ori R6K ; *parSI*-FRT-Rif-FRT-*parSI* between homologies around L3I ; CmR, Rif | This study |
| pPOS228 | pUC vector ; oriC-zeoR between homology zones around L3I ; AmpR, zeoR | This study |
