# Supplementary material for: The Two Cis-Acting Sites, parS1 and oriC1, Contribute to the Longitudinal Organisation of Vibrio cholerae Chromosome I
Source: PLoS Genet. 2014 Jul 10;10(7):e1004448. doi: 10.1371/journal.pgen.1004448 (PMC4091711; doi:10.1371/journal.pgen.1004448)
Supplement: Text S1 — Supplementary Material and Methods. (DOCX) [file pgen.1004448.s047.docx]

**Supporting information :**

**Construction of V. cholerae strains :**

See also Table S1. Marker Frequency Analysis done by deep sequencing revealed the inversion between two *rrn* operons separated by a region of about 150kb containing the origin around 30kb on one side. On the maps, the different tags were positioned with respect to this inversion, but in the table the coordinates of the tags were kept with respect to the N16961 GenBank sequence. Genes coding for fusion proteins LacImcherry and YGFP-ParBT1 were introduced by integration-excision at the *lacZ* locus under the *E. coli lacZ* promoter, using plasmid pAD19.

A library of the N16961 *V. cholerae* strain was built using a mariner transposon to integrate the *tetR* gene using tetracyclin resistance as a selection. The insertion was mapped in 100 of the clones by direct sequencing of the DNA flanking the point of insertion by arbitrary-random PCR [1]. Out of this collection, we chose clones in which the *tetR* insertions occurred outside an open reading frame and outside an obvious promoter. These insertions were checked by PCR and their unicity by southern blot. While *tetR* confered sufficient resistance for the construction of the library, it failed as a selective marker for natural transformation. Therefore, we inserted the *zeo* gene within the *tetR* sequence of each of the clones of interest of our library using pGD162. Resistance to zeocin could then be used to recover the positions into ADV7 by natural transformation. Positions we could not obtain by transposition were engineered by building a plasmid with tet-zeo-tet between homologies of the zone of interest and adding the position by natural transformation. The *zeo* gene was then replaced by a LacO array associated with a Kanamycin resistance by natural transformation of plasmid pAD20 or by parST1 sites associated with Chloramphenicol resistance by natural transformation of the plasmid pAD39.

ΔparSI strains were made by natural transformation of plasmid pPOS169. New parSI sites at 300 and 490 kb from oriI were inserted by natural transformation of plasmid pPOS184 or for the position at 650 kb from oriI, by natural tranformation of plasmid pPOS185. The ectopic oriI was added by natural transformation of plasmid pPOS228.

See plasmids in Table S2.

**Deletion of *oriCI* :**

After introducing an ectopic *oriCI* using natural transformation with pPOS228, we attempted to delete the original Origin of replication of the chromosome I. Two strategies were performed: a direct deletion by replacing the oriCI with a Rif resistance gene or an indirect deletion, by first replacing oriCI by an oriCI flanked by FRT sites and secondly deleting it by inducing a Flipase protein (pFLP2, (2)). (See Figure S17)

When the second oriCI was introduced in the middle of the replichore (near L3I locus), we were unable to delete the original oriCI, even thought it was possible to replace it by a FRT-oriCI-FRT. It was however possible to delete this Origin when the second oriCI was at only 50 kb from it.

**Natural transformation :**

Receiving strains were streaked on LB-agar plates supplemented with antibiotics and left to grow at 37˚C overnight. A preculture on 5mL of LB + antibiotics was then started for each strain, until OD ≈ 0.6 . 1mL of culture was centrifugated at 5000rpm during 10 min, and the pellet was resuspended in the same volume of M9 media. Chitine flakes were inoculated with 1mL of washed cells and 1mL of fresh M9 media and left at 30˚C overnight. The next day, the biofilms were centrifugated at 5000rpm during 10min. Supernatant is removed and replaced by 1mL of fresh M9. The DNA containing the mutation to insert on the genome of the receiving strain is added to the biofilm which is left at 30˚C during 3h to overnight. The biofilm is vortexed and different concentrations of supernatant are plated on LB-agar + antibiotics.

**Microscopy :**

Cells were first grown in 5 ml LB media supplemented with antibiotics at 37˚C until a density of ≈ 0.6 OD_600_ units, then diluted 1/100 in 5 ml M9 media and grown at 37˚C until a density of ≈ 0.6 OD_600_ units. The cultures are then diluted 1/1000 in 5 ml M9 media for an overnight growth at 25 ˚C. The next day, the cultures were diluted ¼ times in 5 ml M9 media and grown at 37˚C, until a density of ≈ 0.2 OD_600_ units.

For snapshots, an agar pad (1% in M9) was cast on a slide. 4μl of cell culture was allowed to adsorb onto the pad before addition of a coverslip. Cells were vizualised using a DM6000-B(Leica) micorscope.

For images of timelapse, a Geneframe(Thermo Scientific) was glued on a slide, and a thin pad of agarose (1% in M9) was cast into the Geneframe. After polymerisation, a 3mm large part of the pad was cut in the middle to create a chamber allowing renewal of oxygen in the pad. 4μl of cell culture was allowed to adsorb onto the pad before addition of a coverslip. Cells were visualized with a microscope Axio Observer (Zeiss) coupled with a CSU 1x Spinning Disk, a 491 laser illumination line and a EVOLVE 512 EM-CCD camera (Roper scientific) at a constant temperature of 30˚C. Images were analysed using the Metamorph software.

Ref :

(1) : O'Toole GA, Kolter R (1998) Initiation of biofilm formation in Pseudomonas fluorescens WCS365 proceeds via multiple, convergent signalling pathways: a genetic analysis. *Mol Microbiol* **28:** 449-461

# (2) : Hoang TT, Karkhoff-Schweizer RR, Kutchma AJ, Schweizer HP (1998) A broad-host-range Flp-FRT recombination system for site-specific excision of chromosomally-located DNA sequences: application for isolation of unmarked Pseudomonas aeruginosa mutants. *Gene* 28 : 77-86
